# SOURCES OF BURDEN IN CAREGIVERS OF PERSONS WITH EARLY- AND LATE-ONSET ALZHEIMER’S DISEASE

**DOI:** 10.1093/geroni/igad104.2558

**Published:** 2023-12-21

**Authors:** Adele Crouch, Lauren Massimo

**Affiliations:** Indiana University School of Nursing, Indianapolis, Indiana, United States; University of Pennsylvania, Philadelphia, Pennsylvania, United States

## Abstract

Functional impairment and neuropsychiatric symptoms (NPS) are common in Alzheimer’s disease (AD) and contribute to caregiver burden. Persons with early-onset AD (EOAD) on average experience greater functional impairment and NPS compared to late-onset AD (LOAD), yet their contribution to caregiver burden have not been explored. Objectives were to: 1) compare functional impairment, NPS, and caregiving burden in EOAD and LOAD; 2) identify sources of caregiver burden including functional impairment and NPS in EOAD and LOAD. Caregivers of 85 persons with EOAD (n=63) and LOAD (n=22) completed questionnaires [Functional Activities Questionnaire, Neuropsychiatric Inventory, Zarit Burden Inventory]. T-tests compared group differences in function, NPS, and caregiver burden. Persons with EOAD had greater NPS frequency than LOAD (t= 2.275, p=0.026). There were no significant differences between groups in function or caregiver burden. Multivariate regression analyses were performed in AD groups, with function and NPS frequency as predictors of caregiver burden covarying for age, caregiver sex, and global cognitive function (MMSE). In the total AD sample, the model explained 12.6% of the variance in caregiver burden [F(5,52)=2.64, adjusted R2=0.126; p=0.033] and poor function was a significant predictor (standardized B=0.42, p=0.003) of caregiver burden. In EOAD, the model explained 17% of the variance in caregiver burden [F(5,35)=2.64, adjusted R2=0.17; p=0.04], and poor function was a significant (standardized B=0.42, p=0.015) predictor of caregiver burden; however this was not the case in LOAD. Results suggest poor function predicts caregiver burden in EOAD. Although NPS in EOAD are more frequent, this was not a predictor of caregiver burden.

